# PEG_2000_-DBCO surface coating increases intracellular uptake of liposomes by breast cancer xenografts

**DOI:** 10.1038/s41598-022-14947-8

**Published:** 2022-06-22

**Authors:** Daxing Liu, Jules Cohen, Nashaat Turkman

**Affiliations:** 1 Stony Brook Cancer Center, Stony Brook, Long Island USA; 2 Department of Radiology and Cancer Center, Renaissance School of Medicine, Stony Brook University, 100 Nicolls Road, Stony Brook, NY 11794 USA; 3 Division of Hematology/Oncology, Department of Medicine, School of Medicine, Stony Brook University, Long Island, NY USA

**Keywords:** Cancer, Oncology, Nanoscience and technology

## Abstract

Given our interest in the utility of liposomes for molecular imaging and theranostics, we investigated how coating the outer layer of the liposome affects internalization by breast cancer cell lines in vitro and in breast tumor tissues in vivo. Indeed, we discovered that a remarkably high liposomal uptake can be achieved by DBCO (dibenzocyclooctyne) soft coating. Our data demonstrates that decorating the terminal lipid with a DBCO moiety at a specific density induces increased tumor uptake in vivo (tumor uptake ~ 50%) compared to conventional undecorated liposome (tumor uptake ~ 20%). In this study, we report improved visualization of breast cancer cells in vivo using a 4T1 orthotopic breast cancer model and primary breast tumor xenograft models MDA-MB-231 and MDA-MB-436. L-PEG_2000_-DBCO coated liposomes demonstrate increased accumulation in breast cancer cells independent of tumor size, type, position, receptor expression, as well as the condition of the host mice. We expect these findings to have a major positive impact on the practical utility of liposomes in image-guided applications and precision medicine theranostics.

## Introduction

Liposomes are lipid vesicles consisting of a lipid bilayer encapsulating an aqueous core and are considered among the most promising and effective drug-delivery vehicles^1,2^. Liposomes have been extensively studied over the past three decades to optimize their clinical potential. Among the most successful applications of liposomes in drug delivery are the two liposomal doxorubicin formulations (Doxil, Myocet) approved for clinical use in ovarian cancer and multiple myeloma, among other diseases^3–5^. However, despite these therapeutic successes, the clinical development of liposomes in molecular imaging and in image-guided theranostics is substantially farther behind. So far, the main limitation to overcome is the relatively low liposomal uptake by tumor tissues. There is an unmet need for new liposomal formulations that can facilitate high tumor uptake in order to facilitate clinical translation of these amazing nanocarriers.

Surface modifications enable the custom design of liposomes for diagnostic, therapeutic, and image-guided delivery applications^6–11^. The unique advantages of liposomes include minimal immune reactivity, reduced proteolytic degradation, increased circulation times and reproducible assembly in a cost-effective manner. As a result, liposomes are almost magical nanocarrier tools for diagnosis, monitoring and management of human disease^12,13^.

Given our interest in the clinical development of liposomes for molecular imaging theranostics, we investigated how coating the outer layer of the liposome at a specific surface density affects cellular internalization in vitro and in vivo. Indeed, we discovered that remarkably high liposomal uptake by tumor tissues can be achieved in vitro and in vivo by DBCO (dibenzocyclooctyne) soft coating. Our data shows that decorating the terminal lipid with a DBCO moiety at specific density produces profound tumor uptake in vivo (~ 50%) compared to the traditional undecorated liposome (tumor uptake ~ 20%). In an animal model, we were able to visualize increased uptake by 4T1 orthotopic breast cancer cells and xenografts from primary breast cancer cell lines MDA-MB-231 and MDA-MB-436. Our findings are consistent with recent findings that interactions between the liposomal surface and the cell membrane significantly influences cellular uptake. Better understanding of how the liposomal surface regulates cell internalization pathways presents an opportunity for improved intracellular drug delivery^14^.

Our findings will pave the way for the development of the next generation of liposomes with modified surface properties that facilitate efficient tumor uptake and, in turn, demonstrate immense potential for clinical use in precision medicine theranostics. We expect to harness the advantages of these enhanced liposomes for precision image-guided surgery, precision detection of tumor with positron emission tomography (PET) radiotracers and tumor treatment via precise delivery of radiotherapeutic payload to the primary tumor and its distant metastases. These ambitious studies are ongoing in our laboratory and will be published in due course.

## Results

### Liposome assembly

**Figure 1:**
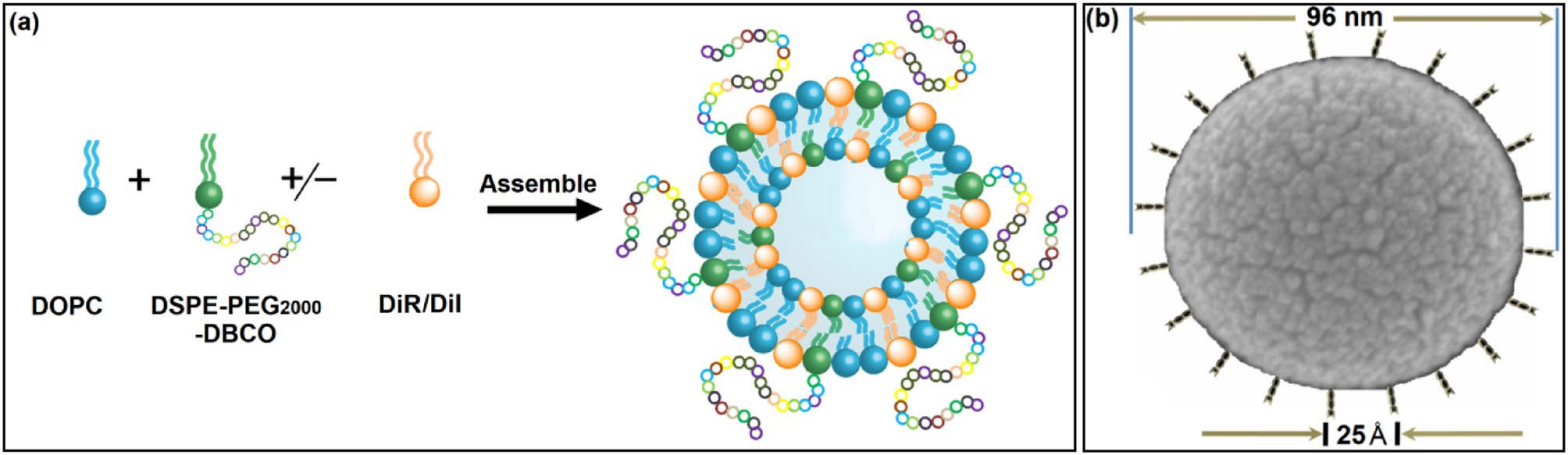
L-PEG_2000_-DBCO assembly with desirable DBCO density: (**a**) scheme of liposomal assembly procedure and (**b**) L-PEG_2000_-DBCO surface morphology was imaged using scanning electron microscopy (SEM) and the DBCO coating array is depicted by a carton scheme to show the potential structure of L-PEG2000-DBCO surface morphology.

The liposomes were assembled as illustrated in Fig. 1a following these steps: skeleton lipids (DOPC), functional lipids (DSPE-PEG_2000_-DBCO, or DSPE-PEG_2000_) and imaging materials (DiI, or DiR) were dissolved into chloroform and formed the thin film followed by rehydration, extrusion and dialysis. The size and zeta potential were ~ 95 nm and − 4.8 mV respectively (Table S1). L-PEG_2000_-DBCO surface morphology was imaged using scanning electron microscopy (SEM) as shown in Fig. 1b. L-PEG_2000_-DBCO (L-DBCO) diameter was approximately 96 nm with 25 Å distance between adjacent DBCO terminals (Fig. 1b and Table S1).

### Liposomal uptake in normal and breast cancer cells by flow cytometry and confocal microscopy demonstrate remarkable L-PEG_2000_-DBCO uptake in cancer cells

**Figure 2:**
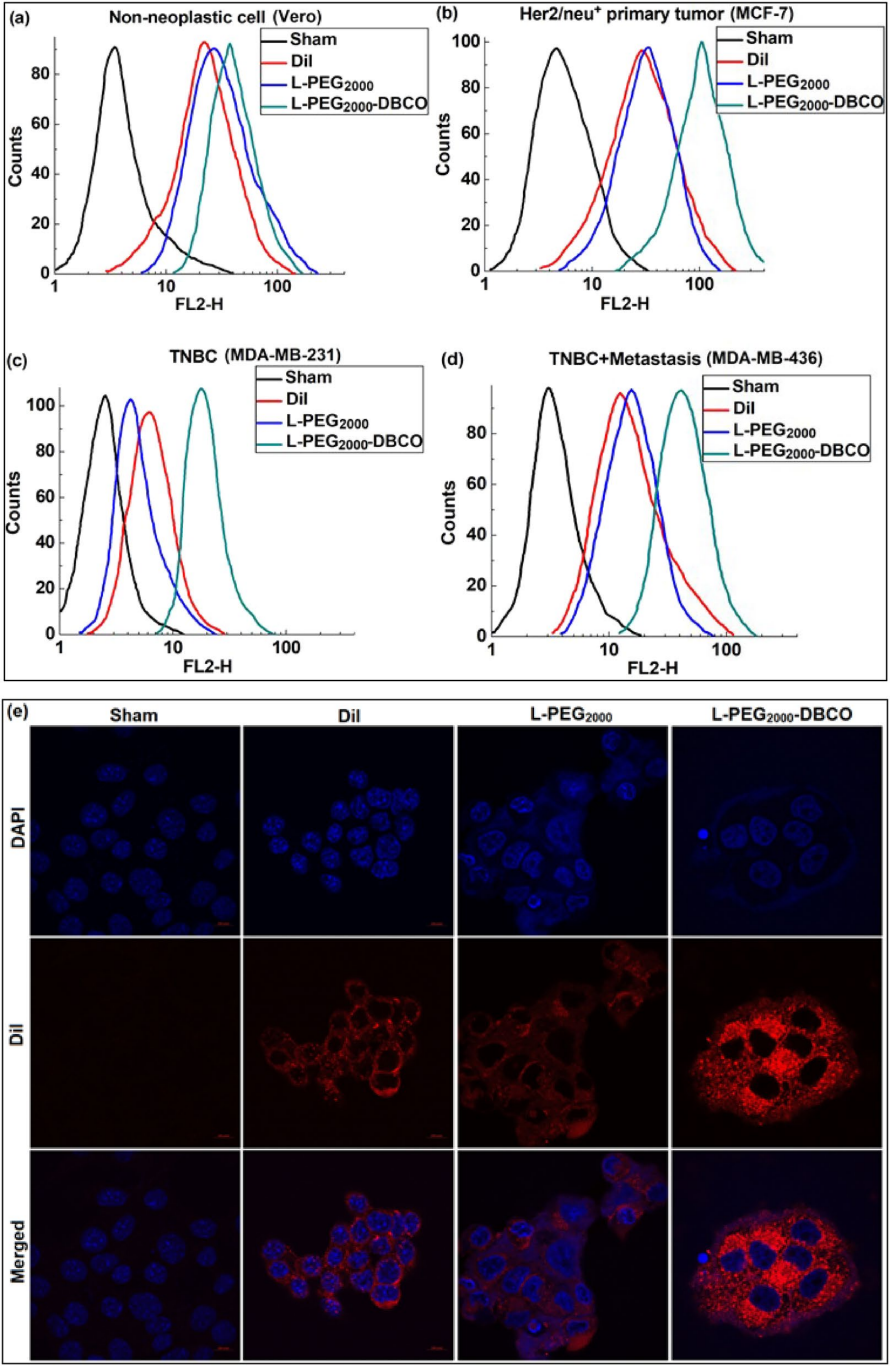
Flow cytometry and confocal microscopy studies using liposomal formulations labeled with DiI (except that sham represent cultured medium): (**a**) non-neoplastic cell line (Vero), (**b**) Her2/neu^+^ primary tumor cell line (MCF-7), (**c**) Triple negative breast cancer (TNBC) cell line (MDA-MB-231), (**d**) Triple negative breast cancer (TNBC) cell line with high metastatic potential (MDA-MB-436) and (**e**) Confocal microscopy of liposomal formulations in MDA-MB-231 cell lines.

We utilized flow cytometry and confocal microscopy in vitro to demonstrate that L-PEG_2000_-DBCO is superior to the conventional L-PEG_2000_. As expected, no significant differences in cellular uptake were observed by flow cytometry among L-PEG_2000_ (labeled with DiI) and L-PEG_2000_-DBCO (labeled with DiI) in non-neoplastic cell line (Vero) as shown in Fig. 2a. In contrast, L-PEG_2000_-DBCO displayed higher cellular uptake in breast cancer cells: MCF-7, MDA-MB-231and MDA-MB-436 as shown in Fig. 2b–d. The mean value of DiI, L-PEG_2000_ and L-PEG_2000_-DBCO peaks to different cells were listed in Table S1 (supplementary material). The signal intensity of L-PEG_2000_-DBCO staining increased by 258%, 303%, 255% in MCF-7, MDA-MB-231 and MDA-MB-436 respectively compared to L-PEG_2000_. Taken together, these data demonstrates that both liposomal formulations (labeled with DiI) distinguished normal cells from breast cancer cells and L-PEG_2000_-DBCO is superior at accumulating in breast cancer cell compared to L-PEG_2000_. Moreover, we used confocal microscopy to further confirm our findings. The staining intensity of L-PEG_2000_-DBCO increased by 244% in MDA-MB-231 cells relative to L-PEG_2000_ as shown in Fig. 2e.

### Liposomal uptake in small high-grade neoplasia foci

**Figure 3:**
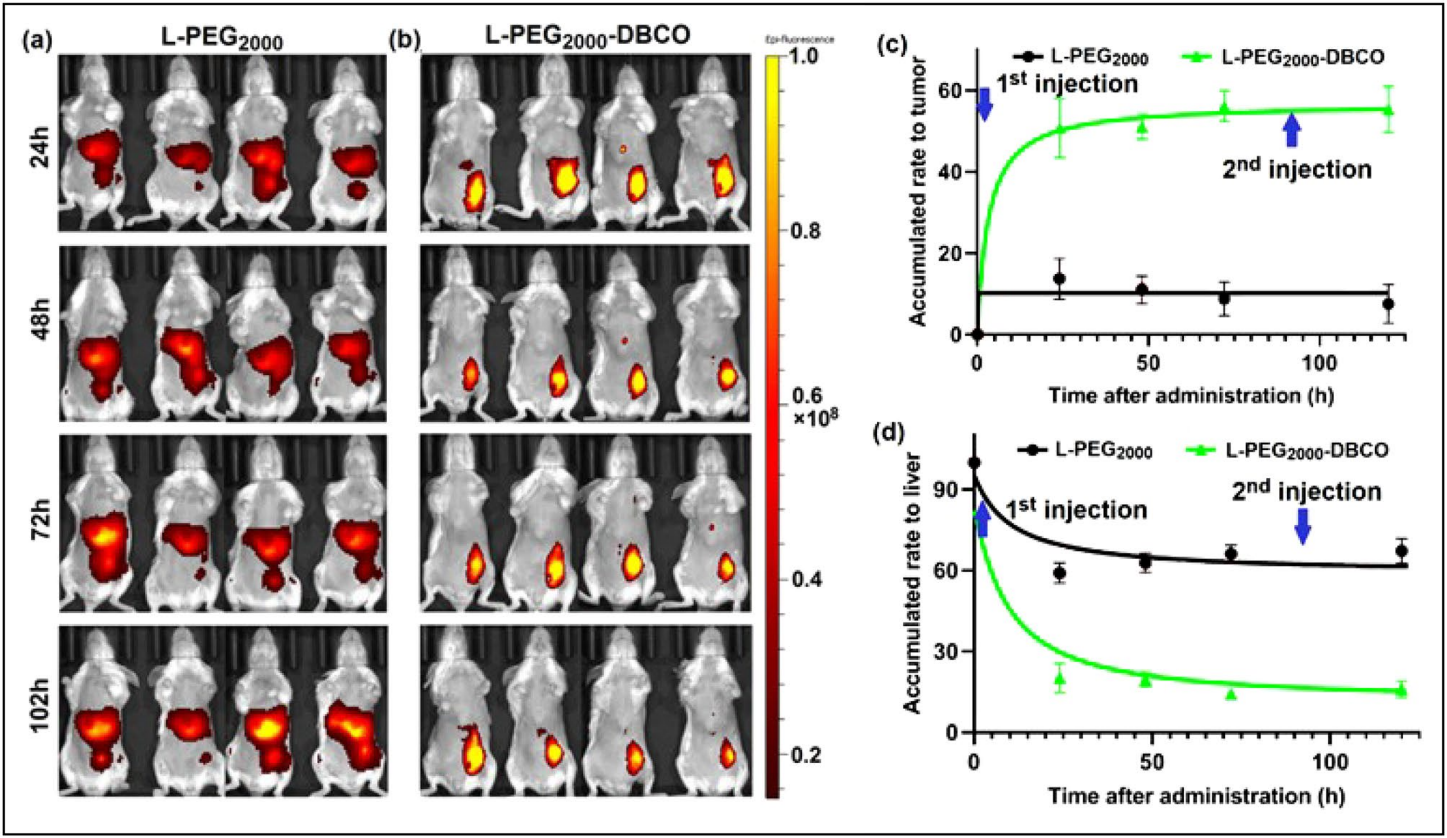
Detection of small allogeneic breast cancer foci (4T1 with tumor size 15 ~ 25 mm^3^) with L-PEG_2000_-DBCO. (**a**,**b**) side-by-side comparison of tumor uptake obtained by (**a**) L-PEG_2000_ and (**b**) L-PEG_2000_-DBCO; (**c**) time course of the biodistribution of L-PEG_2000_ and L-PEG_2000_-DBCO in the tumor over 120 h and (**d**) time course of the biodistribution of L-PEG_2000_ and L-PEG_2000_-DBCO in the liver over 120 h.

We utilized optical imaging in vivo to further confirm and build on the in vitro data above. We tested the performance of L-PEG_2000_-DBCO and L-PEG_2000_ in a small orthotopic tumor (15 ~ 25 mm^3)^ to mimic the metastatic foci or the relapse foci after surgical resection^16,17^. The tumor growth and the metastatic spread of 4T1 cells in BALB/c mice was reported to mimic stage IV human breast cancer^18^. Indeed, the data shown in Fig. 3a–c demonstrates superior tumor uptake of L-PEG_2000_-DBCO compared to L-PEG_2000_ liposome. The L-PEG_2000_-DBCO exhibits remarkably high uptake in tumor foci, as high as 54%, while liver uptake was limited to 16%. In contrast, the L-PEG_2000_ failed to detect this small tumor with 77% the liposome entrapped by liver and the ER system (Fig. 3c,d). The accumulation rate of the two liposomal formulations in tumor tissues is illustrated in Fig. 3c,d. Overall, the L-PEG_2000_-DBCO displayed 700% increased tumor accumulation over the L-PEG_2000_.

Moreover, a second liposomal administration after 96 h did not cause accelerated blood clearance. It has been reported repeated administration PEG-conjugated substances including PEGylated liposomes can cause immunogenic response resulting in the increased clearance and reduced efficacy of PEG-conjugated substances/PEGylated nanocarriers^19–21^.

### Optical imaging with L-PEG_2000_-DBCO displayed high accumulation in vivo in breast cancer xenografts

**Figure 4:**
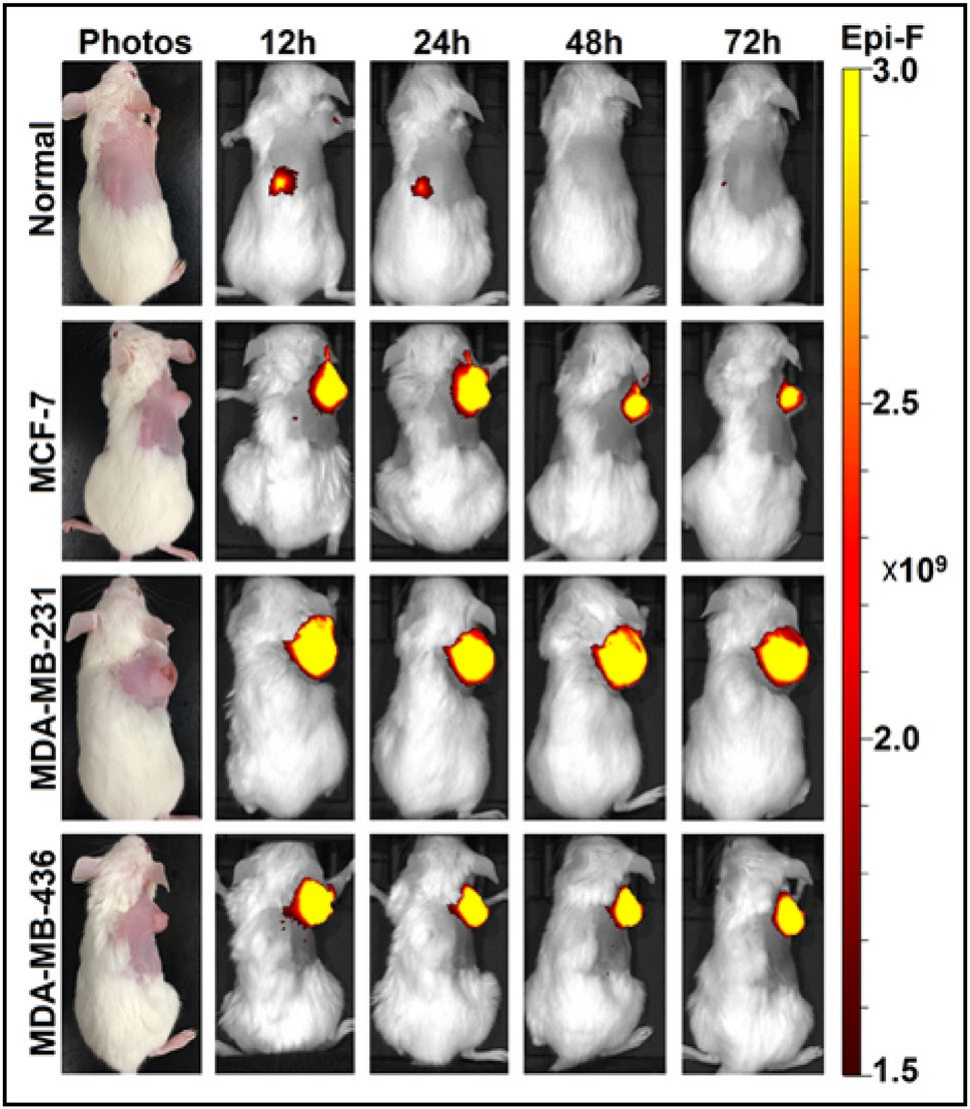
Optical imaging of breast cancer xenografts with L-PEG_2000_-DBCO: MCF-7 (211 mm^3^), MDA-MB-231 (507mm^3^) and MDA-MB-436 (232 mm^3^).

We further extended or studies to other breast cancer xenografts and further confirmed the ability of L-PEG_2000_-DBCO to detect breact tumor xenografts. As shown in Fig. 4 L-PEG_2000_-DBCO accumulated with high concentration in MCF-7 (211 mm^3^), MDA-MB-231 (507 mm^3^) and MDA-MB-436 (232 mm^3^) breact tumor xenografts.

**Figure 5:**
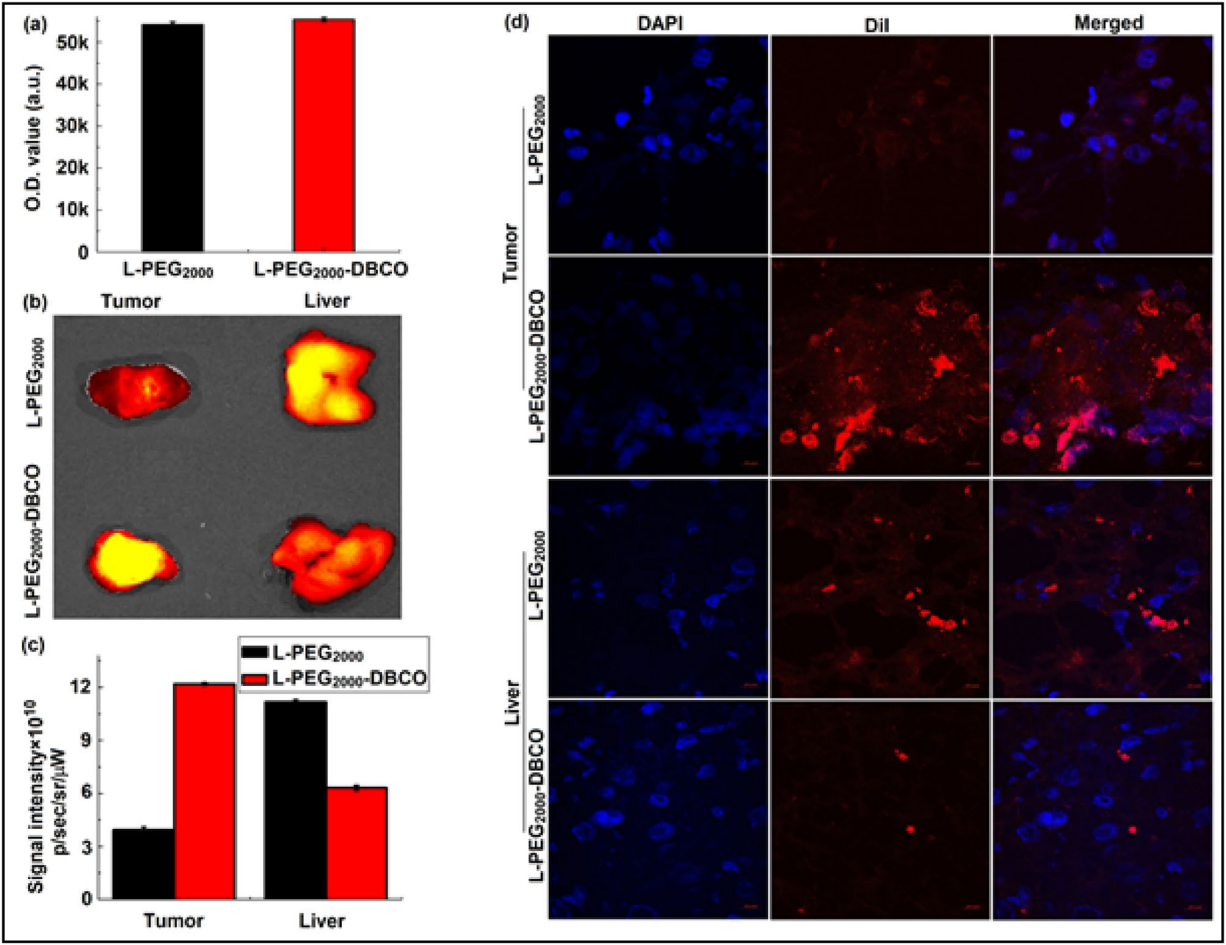
Ex vivo optical imaging and confocal microscopy with L-PEG_2000_-DBCO and L-PEG_2000_ in tumor tissues and liver of MDA-MB-231 tumor xenograft: (**a**) control experiment of demonstrating equal fluorescence intensity of L-PEG_2000_ and L-PEG_2000_-DBCO, (**b**) ex vivo optical imaging with L-PEG_2000_-DBCO and L-PEG_2000_ in tumor tissues and the liver, (**c**) quantitative uptake based on fluorescent signal intensity of L-PEG_2000_-DBCO and L-PEG_2000_ in tumor tissues and the liver at 24 h post liposome administration and (**d**) confocal microscopy imaging of L-PEG_2000_-DBCO and L-PEG_2000_ in tumor tissue and liver sections.

We then excised liver and tumor tissues from the mice with MDA-MB-231 tumor xenograft and performed ex vivo optical imaging and confocal microscopy. The results were also consistent with data above confirming the superiority of L-PEG_2000_-DBCO over L-PEG_2000_. As shown in Fig. 5, L-PEG_2000_-DBCO outperformed L-PEG_2000_ by over three folds in tumor and accumulated 40% less in the liver (Fig. 5b,c). Moreover, confocal microscopy also demonstrated higher tumor uptake and lesser uptake for L-PEG_2000_-DBCO compared to L-PEG_2000_ (Fig. 5d). Altogether, the in vivo and the ex vivo results strongly support the high performance of L-PEG_2000_-DBCO at accumulating with high concentration in tumor tissues which underscores the immense potential for the utility of L-PEG_2000_-DBCO as a powerful nanocarrier of various molecular imaging and image-guided applications.

### L-PEG_2000_-DBCO demonstrated remarkable ability at visualizing small tumors

**Figure 6:**
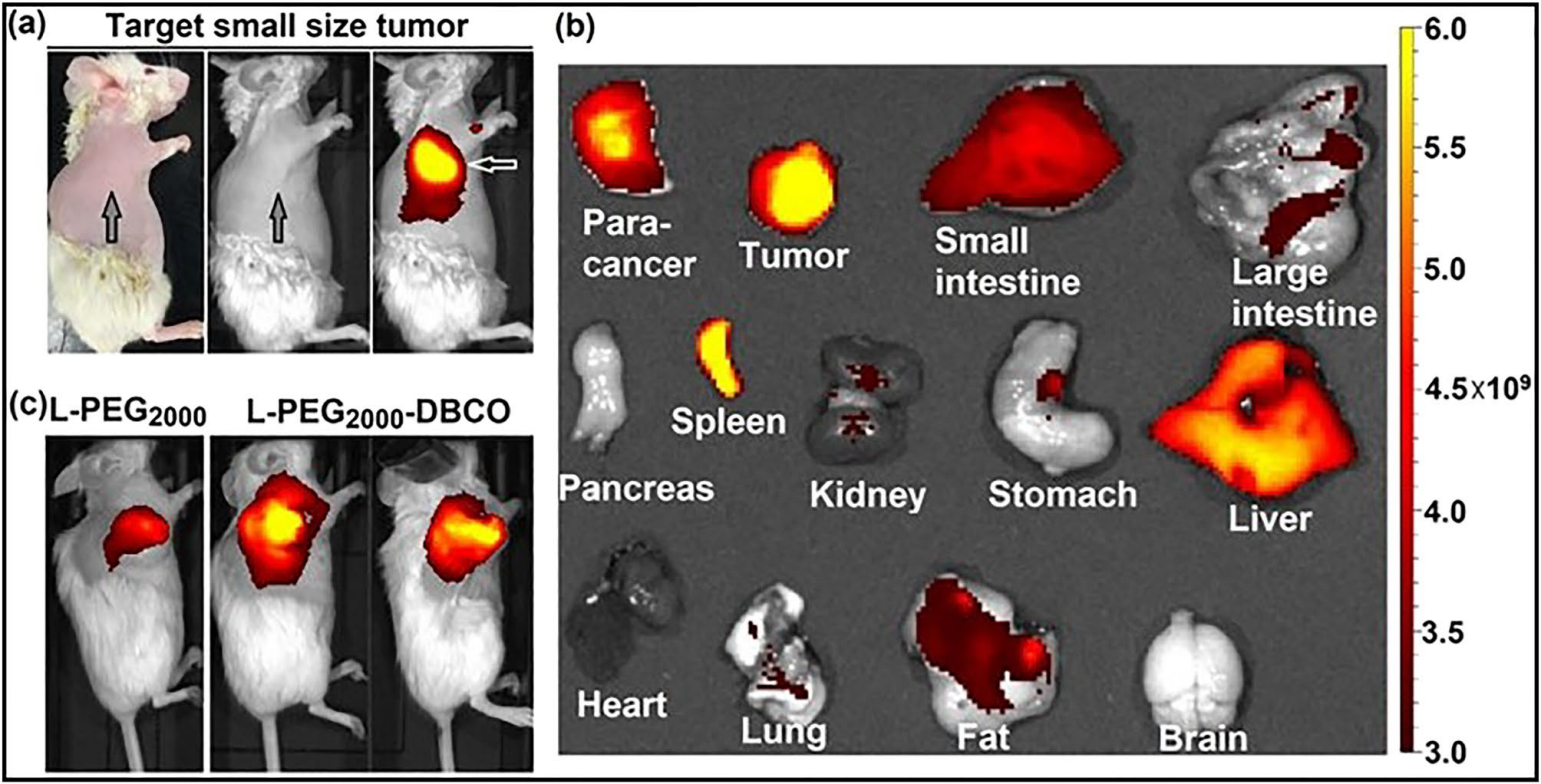
(**a**) Optical imaging with L-PEG_2000_-DBCO enabled detection of early stage of subcutaneous breast tumor (MDA-MB-231 with tumor size 10 ~ 20 mm^3^, 3 days after implantation, invisible to both naked eye and bright field as indicated by arrows); (**b**) ex vivo optical imaging of the tumor and major organs with L-PEG_2000_-DBCO and (**c**) optical imaging of MDA-MB-231 tumor xenograft with L-PEG_2000_ compared with L-PEG_2000_-DBCO.

To finalize and strengthen our findings, we examined the utility of L-PEG_2000_-DBCO in vivo to demonstrate the remarkable ability of L-PEG_2000_-DBCO at visualizing small tumors. Indeed, L-PEG_2000_-DBCO was able to detect small subcutaneous breast tumor (MDA-MB-231, size 10 ~ 20 mm^3^) which was invisible to both the naked eye and the bright field as indicated by arrows in Fig. 6a,b. It is important to note, that the small tumor is implanted in the fat pad and therefore it was hard to isolate from the para-cancer tissue. As a result, the tumor size in Fig. 6b appears to be larger than the accurate size of the solid tumor as was measured by the caliper (photo is shown in Figure S4). Also, Fig. 6b indicates that our L-PEG2000-DBCO has a super targeting capacity on both cancer tissue and para-cancer tissue as the signal of para-cancer tissue is shown in Fig. 6b. Furthermore, L-PEG_2000_-DBCO was superior to L-PEG_2000_ in tumor xenograft with larger size as shown in Fig. 6b. Ex vivo optical imaging also confirmed the in vivo data as shown Fig. 6c.

## Discussion

Despite significant work on liposomal technology over the last several decades, approaches to optimize the surface formulation of liposomes have remained largely unexplored. Our findings demonstrate a key role for surface functional moieties in cellular internalization and tumor uptake. Liposomal surfaces have been extensively utilized to conjugate drugs (the so-called targeted liposome) for various therapeutic applications, some using the DBCO moiety as the site for drug conjugation through copper free “click chemistry”^22–24^. However, surface conjugation may reduce the efficiency of liposomal cellular internalization and tumor uptake thus reducing the effectiveness of drug delivery. In this study, we discovered that coating liposomal surface with the DBCO moiety (L-PEG_2000_-DBCO) can lead to remarkably high tumor uptake. We performed a series of in vitro, and in vivo and ex vivo experiments to demonstrate that L-PEG_2000_-DBCO is superior to conventional L-PEG_2000_. We initially studied liposomal uptake in normal and breast cancer cells in vitro by flow cytometry and confocal microscopy. Flow cytometry indicated higher L-PEG_2000_-DBCO uptake compared to L-PEG_2000_, a finding which was further confirmed by confocal microscopy (Fig. 2). Since optical imaging can be used effectively to monitor liposomal trafficking in vitro and in vivo via the fluorescent dye encapsulated in the liposomal lipid bilayer, we utilized optical imaging to track the biodistribution of L-PEG_2000_-DBCO in vivo in various models of breast cancer. In a control experiment, liposome solutions with equal volumes of L-PEG_2000_ and L-PEG_2000_-DBCO exhibited similar fluorescence intensity (Fig. 5a and Figure S2), justifying use of fluorescence intensity as a measure to quantify liposomal biodistribution in vitro and in vivo and ex vivo. Examination of breast tumors (Figs. 3, 4, 5, 6) demonstrated that L-PEG_2000_-DBCO was preferentially taken up in vivo by tumor tissues and reduced uptake in liver and spleen when compared to L-PEG_2000_. For example, the in vivo data obtained with MDA-MB-231 tumor xenograft showed that the tumor signal intensity of the L-PEG_2000_-DBCO and L-PEG_2000_ were 12.2 × 10^10^ and 3.9 × 10^10^ p/sec/sr/mW (Fig. 5c), respectively, corresponding to 213% increase in L-PEG_2000_-DBCO tumor accumulation when compared to L-PEG_2000_. In contrast, the liver signal intensity of the L-PEG_2000_ and L-PEG_2000_-DBCO were 11.2 × 10^10^ and 6.3 × 10^10^ p/sec/sr/mW, respectively corresponding to 46% reduction in liver uptake of L-PEG_2000_-DBCO relative to L-PEG_2000_. Moreover, tumor and liver tissue sections imaged by confocal microscopy further confirmed the in vivo and the ex vivo optical imaging (Fig. 5d).

We expect our findings to find application in the efficient delivery of molecular imaging compounds, image-guided probes, and cancer therapeutics. One advantage is the ability of L-PEG_2000_-DBCO to visualize small tumors such as a small orthotopic 4T1implant (Fig. 2). L-PEG_2000_-DBCO was able to detect a small subcutaneous breast tumor (MDA-MB-231, size 10 ~ 20 mm^3^) which was invisible to both the naked eye and the bright field as indicated by arrows in Fig. 6. Taken together, the data in Figs. 3 and 6 demonstrate that L-PEG2000-DBCO has shown the capacity to visualize the small tumor in both allogeneic (4T1) and xenogeneic (MDA-MB-231) transplantation models. Furthermore, L-PEG_2000_-DBCO was able to detect, visualize and discriminate between a small tumor (10 ~ 15 mm^3^) and the adjacent main MDA-MB-231 tumor (196 ~ 255 mm^3^) and to accumulate in the small tumor with the same signal density of that of the large tumor (Figure S3). Therefore, we are currently pursuing the utility of L-PEG_2000_-DBCO for molecular imaging and image-guided surgery.

### Future perspective

The mechanism that drives the high tumor uptake of L-PEG_2000_-DBCO and how this is facilitated by the DBCO moiety is not fully understood. For example, conjugation of L-PEG_2000_-DBCO with DV1-N3 peptides leads to diminished tumor uptake, similar to L-PEG_2000_, underscoring the key role of the DBCO moiety in driving high tumor uptake^12^. It is likely that the enhanced permeability and retention (EPR) effect contributes to enhanced tumor uptake. Long circulation times allow the liposome to penetrate preferentially into tumor tissue through permeable tumor vasculature and to remain in the tumor bed through impaired lymphatic drainage^25^. However, the EPR effect alone was reported to offer less than a twofold increase in nano-drug delivery^25,26^. Further studies of DBCO-labeled liposomes will be needed to help unravel the mechanism of high accumulation of L-PEG_2000_-DBCO in tumor tissues and low accumulation in non-target tissues that ordinarily mediate therapeutic toxicity. Enhanced chemical modifications of DBCO-labelled liposomes may lead to enhanced formulations with improved tumor specificity. These studies are ongoing in our laboratory and will be reported in future publications.

In summary, the liposome surface may significantly influence in vitro cellular uptake and in vivo penetration into tumor tissues while minimizing penetration into off-target tissues. We are optimistic that our findings will pave the way for the design of next generation liposomes for efficient delivery of molecular imaging and image-guided probes as well as anti-cancer therapeutics.

## Conclusion

We performed a series of in vitro and in vivo experiments to demonstrate that liposomal surface modification may play a key role in inducing high cellular internalization in vitro and high liposomal accumulation in cancer tissues in vivo. Specifically, we discovered that a soft layer of DBCO induces a remarkable increase in tumor uptake of L-PEG_2000_-DBCO compared to L-PEG_2000_ despite both liposomal formulations sharing an identical skeleton. We have demonstrated the increased ability of L-PEG_2000_-DBCO to accumulate in breast cancer foci independent of tumor size, type, position, receptor expression, as well as the condition of the host mice. Remarkably, a significant reduction in L-PEG_2000_-DBCO uptake in the liver and off target tissues was also observed compared to L-PEG_2000_. Altogether, our findings will pave the way for the development of new liposomal formulations with enhanced internalization by tumor tissues. We are currently exploring the utility of L-PEG_2000_-DBCO as an effective carrier of molecular imaging and image-guided probes.

## Materials and methods

### Reagents and materials

1,2-distearoyl-sn-glycero-3-phosphoethanolamine-N-[dibenzocyclooctyl(polyethylene glycol)-2000] (ammonium salt) (DSPE-PEG_2000_-DBCO), 1,2-distearoyl-sn-glycero-3-phosphoethanolamine-N-[methoxy(polyethylene glycol)-2000] (ammonium salt) (DSPE-PEG_2000_) and 1,2-dioleoyl-sn-glycero-3-phosphocholine (DOPC) were purchased from Avanti Polar Lipids (Alabaster, AL). 1,1′-Dioctadecyl-3,3,3′,3′-tetramethylindocarbocyanine perchlorate (DiI) was purchased from Sigma-Aldrich (St. Louis, MO). Cy5 amine (non-sulfonated) was purchased from APExBIO Technology LLC (Houston, Texas). (1,1'-Dioctadecyl-3,3,3',3'-Tetramethylindotricarbocyanine Iodide) (DiR) was purchased from Biotium™ (Fremont, CA). Certified Fetal Bovine Serum (FBS) was obtained from Gibco® by Life Technologies Corporation (Grand Island, NY). Nuclepore track-etched membrane (Pore size: 100 nm, 200 nm) was obtained from Whatman (Florham Park, NJ).

### Liposome assembly

Liposome modified with PEG_2000_-DBCO (L-PEG_2000_-DBCO) and liposome coated with PEG_2000_ (L-PEG) were prepared by the extrusion method^15^. Briefly, a mixture of DOPC: DSPE- PEG_2000_-DBCO: DiR dye (97:2:1, mol:mol:mol) or DOPC: DSPE-PEG_2000_: DiR dye (97:2:1, mol:mol:mol) were solubilized in chloroform and dried in a rotary evaporator under reduced pressure. Notably, the DiR dye can be switched to DiI and Cy5-amine according to the desirable experimental design. The lipid film was hydrated in 5 mL DI water (pH 7.0) with gentle shaking to yield a 2.6 mM lipid solution. The lipid solution went through 10 cycles of freeze–thaw to form multilamellar liposomes. Liposomes were extruded via a Northern Lipids Extruder with 200 nm and 100 nm polycarbonate nanoporous membranes sequentially. After extrusion, the liposome solution was dialyzed in Tris–HCl buffer (pH 7.4) using a Slide-A-Lyzer dialysis cassette (MWCO 100 kDa) overnight at room temperature (RT).

Dynamic light scattering (DLS) was used to monitor the integrity, size, and zeta potential of the vesicles during and after the coupling reaction.

### Scanning electron microscopy (SEM)

Liposomes (1 mL, 2.6 μM lipids) were stained with 1% OsO_4_ in 0.1 M PBS in an ice bath for 1 min. The solution was filtered through a 100 nm nuclepore track-etched membrane. The film was dehydrated in a graded series of ethanol (50%–75%–100%–100%) for 15 min at each step. The film was dried by Critical Point Drying according to the manufacturer’s instructions. The film was adhered to the top of the steel disc with conductive tape, sputtered with gold and used for SEM detection.

### Cell culture

All cell lines were obtained from American Type Culture Collection (ATCC, Manassas, VA). The non-neoplastic kidney epithelial cell from Cercopithecus aethiops—kidney normal cell line (Vero), human Her2/neu^+^ primary breast cancer cell line (MCF-7), human triple negative breast cancer cell lines (MDA-MB-NT17631 and MDA-MB-436) and mouse mammary epithelial cancer cell line (4T1) were used in our current studies. All cancer cell lines were cultured in DMEM with 10% FBS and 100-unit penicillin–streptomycin. All cells were maintained at 37 °C in a humidified incubator with 5% CO_2_.

### Liposomal cellular uptake studies

For liposome binding analyzed by flow cytometry, Vero, MCF-7, MDA-MB-231, and MDA-MB-436 (2 × 10^6^) were seeded in a 75 cm^2^ flask for 2–5 days. After reaching 50% confluence, the cells were detached by 0.25% trypsin/0.1% EDTA followed by washing with PBS twice. After blocking with BSA (1%) for 30 min, samples were stained with liposomes with DiI dye for 2 h at 37 °C in a humidified incubator with 5% CO_2_ (0.15 mM of lipids per 10^6^ cells). After washing with PBS twice, the samples were resuspended in 500 μL PBS and evaluated by flow cytometry using a BD LSR II Analyzer (B&D Bioscience, CA).

Liposomal uptake in cells was analyzed by confocal microscopy. MDA-MB-231 cells (2 × 10^5^) were seeded in a Lab-Tek II Chamber Slide System separately with 2 mL medium overnight at 37 °C. Samples were stained with liposomes with DiI dye for 2 h at 37 °C in a humidified incubator with 5% CO_2_ (0.15 mM of lipids per 10^6^ cells). After the medium was removed, cells were rinsed with PBS twice and fixed with 4% formaldehyde in PBS at RT for 10 min. DAPI was used to stain the cell nucleus followed by washing with PBS three times. Cells were examined under a LSM 710 Confocal fluorescent microscope (Zeiss). Digital images were captured and processed with software Image J (NIH).

### Tumor models

All animal experiments evaluated and approved by the Stony Brook University Institutional Animal Care and Use Committee (IACUC). All animal studies were carried out in accordance with guidelines of the approved IACUC protocol and in accordance with ARRIVE guidelines.

24 female NOD.Cg-Prkdc^scid^/J mice (SCID mice) and 12 female Babl/C mice were ordered from the Jackson Laboratory (Bar Harbor, ME). For allogeneic transplantation model, the 4T1 cells were harvested from 3 plates and washed with PBS. Then 20 k cells per mouse of cell suspensions were injected into the second (count from the bottom) mammary fat pat with small incision to establish a single tumor nodule in the site of injection. After 3 days growth, the 4T1 cells implanted into mammary fat pad and formed 15 ~ 25 mm^3^ solid tumor. Then the mice were divided into two groups: the L-PEG_2000_ group and the L-PEG_2000_-DBCO group, which received the liposomal formulation via IV injection, respectively. The tumor signal intensity, body weight and the living condition of mice were recorded with designed time points after liposome administration. The second cycle of the injection was administrated after 96 h of the first liposome administration.

For xenogeneic transplantation models, the MCF-7, MDA-MB-231 and MDA-MB-436 cells were harvested from 12 plates and washed with PBS. Then 50 k cells per mouse of cell suspensions were injected into the right shoulder subcutaneously to construct the xenogeneic breast cancer models. The tumor size reached around 100 mm^3^ after 7 ~ 10 days for MDA-MB-231, 15 ~ 20 days for MDA-MB-36 and 25 ~ 30 days for MCF-7, respectively. Then the different groups of breast cancer models were injected with the same volume of L-PEG_2000_) or L-PEG_2000_-DBCO.

### Optical in vivo and ex vivo imaging

In vivo and ex vivo imaging was performed using IVIS Lumina III system (Perkin Elmer). Briefly, to make sure the fluorescence intensity at the same level, 3 × 100 μL of the L-PEG_2000_ and L-PEG_2000_-DBCO solutions were placed into 96 well plates separately before administration and scanning under DiR near infrared filed via the following parameters: excitation filter 740 nm, emission filter 790 nm, binning 4 or 8, f/Stop 2. These mice administrated the different liposomes were scanned non-invasively three times under anesthesia (2% isoflurane via the vaporizer of the IVIS instrument). After the in vivo analysis, mice were sacrificed immediately. The organs (tumor, liver, heart, lung, spleen, brain, kidney, small intestine, and colon) were collected, and images were acquired using the same parameters as described above. Collected images were analyzed using Living Image 4.3.1 software (Perkin Elmer): ROIs were designed in order to appropriately select each organ and radiant efficiency calculated.

### Cryostat sectioning

The tumor bearing mice were injected the L-PEG_2000_ and L-PEG_2000_-DBCO liposomes with 2% DiI components, respectively. After 24 h of liposome administration, the tumors and livers are harvested and frozen into − 80 °C refrigerator. The frozen samples were further cut to Sections (10 μm) with a cryostat (CM3050 S, Leica, Germany). The sections were stained with DAPI, followed by sealing with mounting oil, and then observed using a confocal laser scanning microscope (Zeiss, LSM 710).

### Statistical analysis

All quantitative experiments were run in triplicates and the results were expressed as mean ± standard deviation, unless indicated otherwise. Statistical analysis of the data was performed by two-way analysis of variance (ANOVA) with Tukey’s post-test. Differences between groups at a level of p < 0.05 were considered statistically significant (*represented) and those at p < 0.01 as highly significant (**represented).

## Supplementary Information

Supplementary Information.

## Data Availability

All data generated or analyzed during this study are included in this published article [and its supplementary information files].
