# Supplementary material for: PEG2000-DBCO surface coating increases intracellular uptake of liposomes by breast cancer xenografts
Source: Sci Rep. 2022 Jun 22;12:10564. doi: 10.1038/s41598-022-14947-8 (PMC9218082; doi:10.1038/s41598-022-14947-8)
Supplement: Supplementary file 1 — Supplementary Information. [file 41598_2022_14947_MOESM1_ESM.pdf]

Supplementary materials

PEG_{2000}-DBCO surface coating increases intracellular uptake of liposomes by breast cancer xenografts

Daxing Liu[1,2], Jules Cohen[1,3] and Nashaat Turkman[1,2*]

[1]Stony Brook Cancer Center, Stony Brook, Long Island, United States

[2]Department of Radiology, School of Medicine, Stony Brook University, Long Island, NY, United States

[3] Department of Medicine, Division of Hematology/Oncology, School of Medicine, Stony Brook University, Long Island, NY, United States

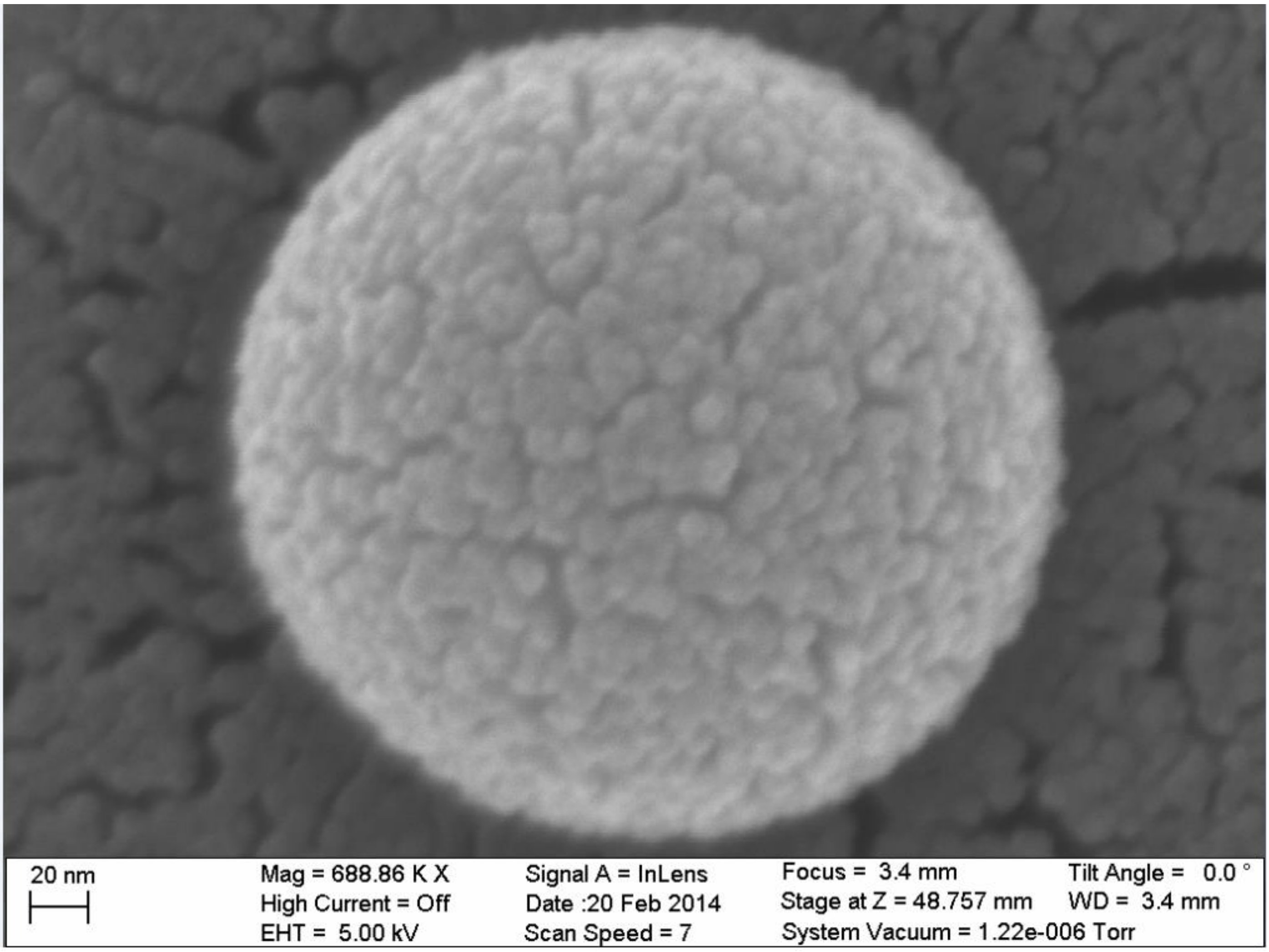

Figure S1. L-PEG_{2000}-DBCO surface morphology obtained by scanning electron microscopy (SEM)

Supplementary Table 1: Size and zeta potential characterization of liposomes.

Liposome	Size (nm)	Zeta (mV)	Number of DBCO per μm^2	Average distance per two DBCO (Å)
L-PEG_{2000}	94.3 ± 1.6	-6.26 ± 0.86	0	0
L-PEG_{2000}-DBCO	96.3 ± 1.7	-3.16± 1.10	45×10^3	25

*The average saturated area per DOPC is 80 Å^2 [34], the measurement unit of the calculated value is DBCO/(μm)^2.

$d=[(A_{DOPC}*C_{Lipid})/(2\pi*C_{DBCO})]^{1/2}$, which is only relative to the concentration of DOPC and DSPE-PEG_{2000}-DBCO.

Control experiment

To ensure and demonstrate equal fluorescence intensity for both the L-PEG2000 and L-PEG2000-DBCO before *in vivo* administration and scanning, 3X100 μL of solutions were placed into 96 well plates separately and the fluorescent intensity was measured near infrared filed via the following parameters: excitation filter 740 nm, emission filter 790 nm, binning 4 or 8, f/Stop 2.

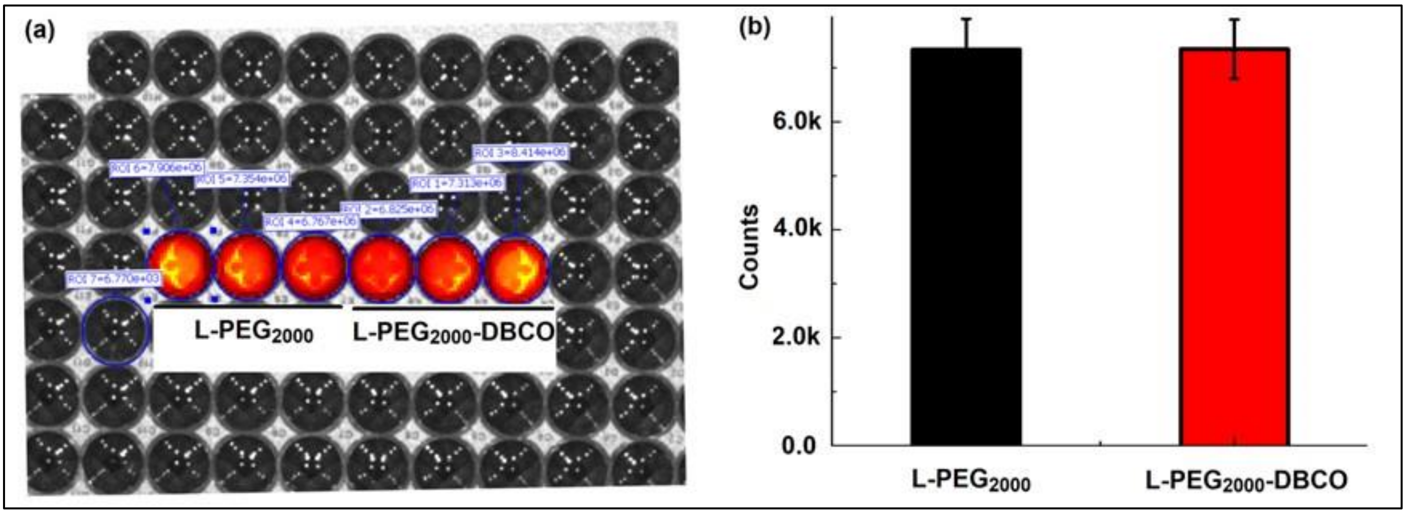

Figure S2. Control experiment (a-b) comparison of the dye concentration of L-PEG_{2000}-DBCO and L-PEG_{2000}.

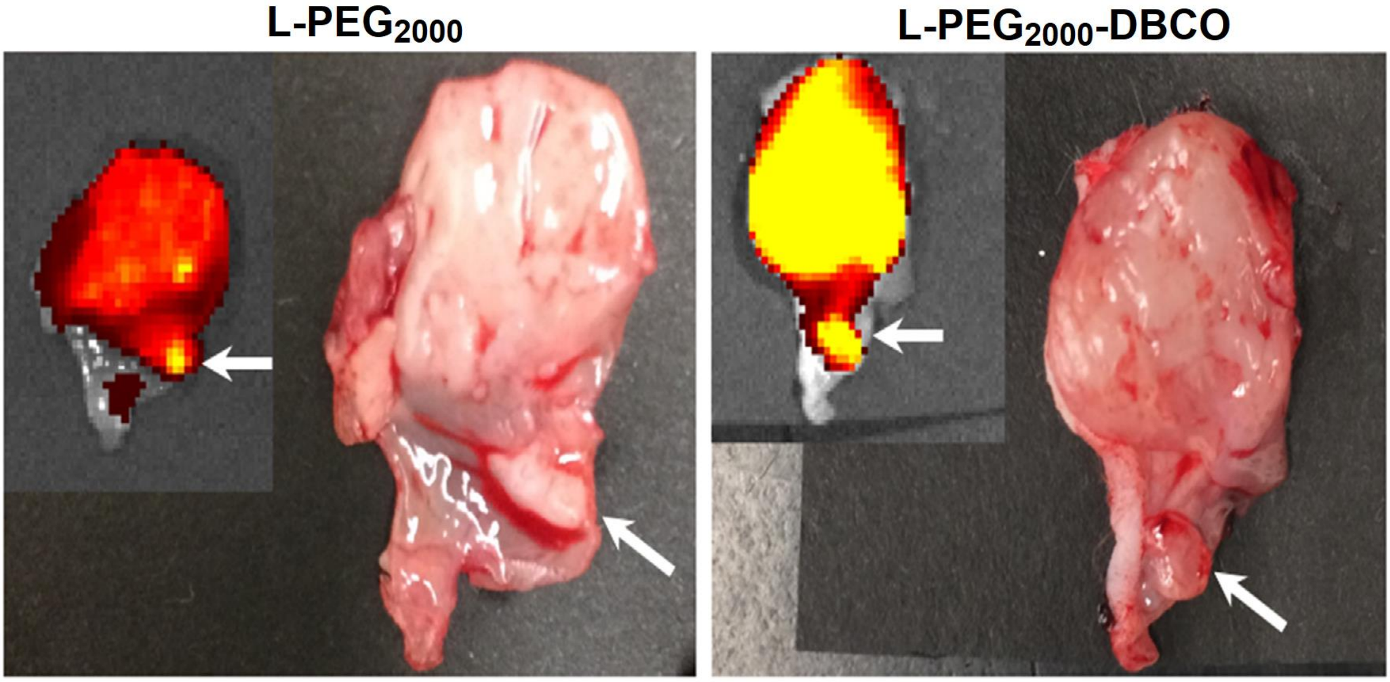

Figure S3. Comparison of the level of accumulation between L-PEG_{2000} and L-PEG_{2000}-DBCO with small size tumor beside the large tumor, thus indicating a high capacity at distinguishing tumor tissues from non-neoplastic tissues.

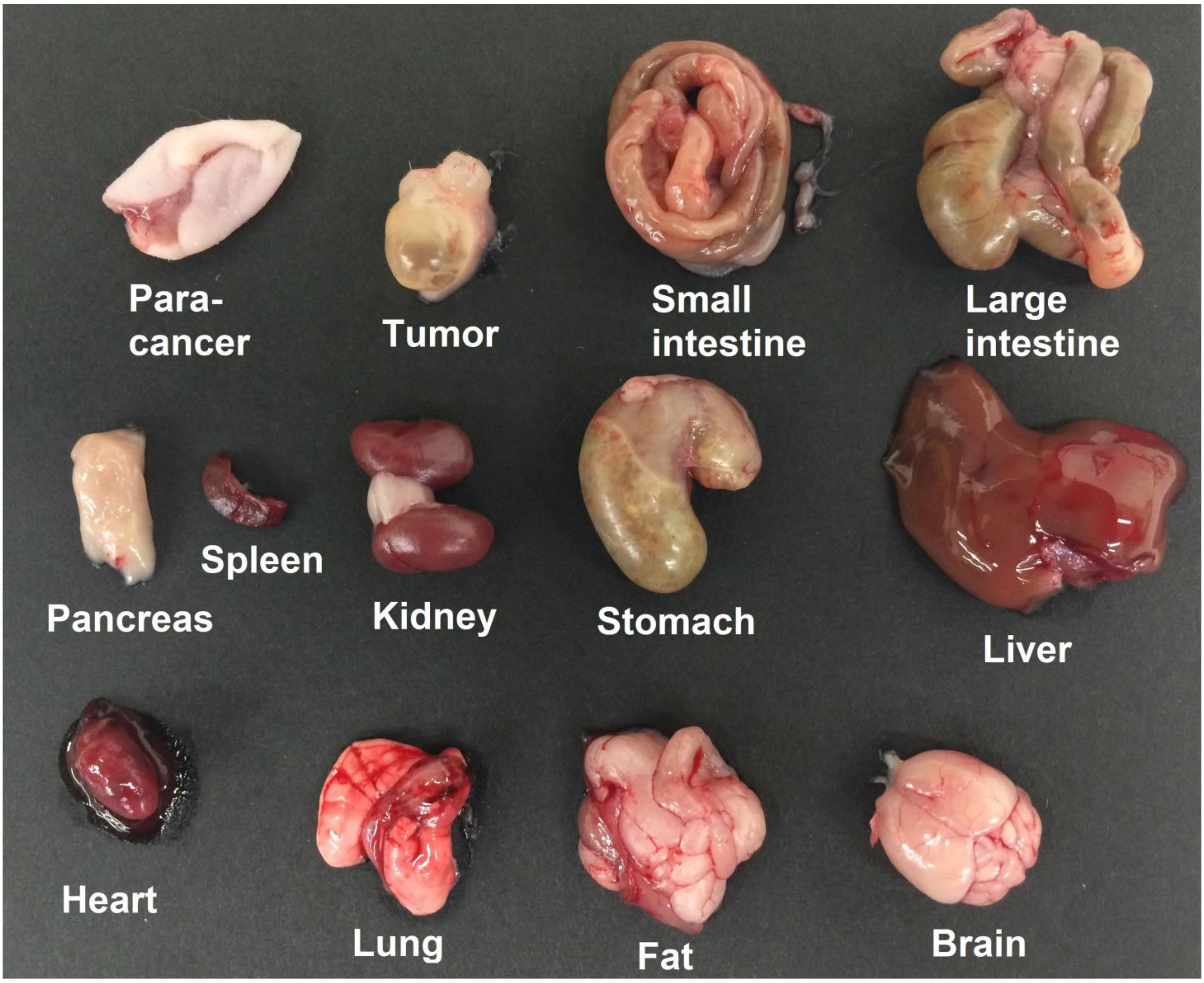

Figure S4. E*x vivo* photograph of the tumor and major organs.
